# Synaptic protein CSF levels relate to memory scores in individuals without dementia

**DOI:** 10.21203/rs.3.rs-4607202/v1

**Published:** 2024-07-23

**Authors:** Kirsten E.J. Wesenhagen, Diederick M. de Leeuw, Jori Tomassen, Johan Gobom, Isabelle Bos, Stephanie J.B. Vos, Pablo Martinez-Lage, Mikel Tainta, Julius Popp, Gwendoline Peyratout, Magda Tsolaki, Rik Vandenberghe, Yvonne Freund-Levi, Frans Verhey, Simon Lovestone, Johannes Streffer, Valerija Dobricic, Kaj Blennow, Philip Scheltens, August B. Smit, Lars Bertram, Charlotte E. Teunissen, Henrik Zetterberg, Betty M. Tijms, Alzheimer’s Disease Neuroimaging Initiative

**Affiliations:** Alzheimer Center Amsterdam, Vrije Universiteit Amsterdam, Amsterdam UMC; Alzheimer Center Amsterdam, Vrije Universiteit Amsterdam, Amsterdam UMC; Alzheimer Center Amsterdam, Vrije Universiteit Amsterdam, Amsterdam UMC; Sahlgrenska University Hospital; Alzheimer Center Amsterdam, Vrije Universiteit Amsterdam, Amsterdam UMC; Alzheimer Center Limburg, Maastricht University; CITA-Alzheimer Foundation; CITA-Alzheimer Foundation; University Hospital of Lausanne; University Hospital of Lausanne; AHEPA University Hospital, Aristotle University of Thessaloniki; University Hospitals Leuven; Karolinska Institutet; Alzheimer Center Limburg, Maastricht University; University of Oxford; University of Antwerp; University of Lübeck; University of Gothenburg; Alzheimer Center Amsterdam, Vrije Universiteit Amsterdam, Amsterdam UMC; VU University Amsterdam; University of Lübeck; Amsterdam Neuroscience, Amsterdam UMC, Vrije Universiteit; Sahlgrenska University Hospital; Alzheimer Center Amsterdam, Vrije Universiteit Amsterdam, Amsterdam UMC

**Keywords:** Synaptic proteins, cerebrospinal fluid proteomics, memory performance, early Alzheimer’s disease

## Abstract

**INTRODUCTION:**

We investigated how cerebrospinal fluid levels of synaptic proteins associate with memory function in normal cognition (CN) and mild cognitive impairment (MCI), and investigated the effect of amyloid positivity on these associations.

**METHODS:**

We included 242 CN (105(43%) abnormal amyloid), and 278 MCI individuals (183(66%) abnormal amyloid) from EMIF-AD MBD and ADNI. For 181 (EMIF-AD MBD) and 36 (ADNI) proteins with a synaptic annotation in SynGO, associations with word learning recall were analysed with linear models.

**RESULTS:**

Subsets of synaptic proteins showed lower levels with worse recall in preclinical AD (EMIF-AD MBD: 7, ADNI: 5 proteins, none overlapping), prodromal AD (EMIF-AD MBD only, 27 proteins) and non-AD MCI (EMIF-AD MBD: 1, ADNI: 7 proteins). The majority of these associations were specific to these groups.

**DISCUSSION:**

Synaptic disturbance-related memory impairment occurred very early in AD, indicating it may be relevant to develop therapies targeting the synapse early in the disease.

## 1. Introduction

Memory impairment is a key feature of Alzheimer’s disease (AD)(Jack et al., 2018; Jansen et al., 2015). Decline in memory function precedes dementia onset, starting already in the preclinical stage of AD (Jansen et al., 2018). A better understanding of mechanisms underlying memory loss can help the development of novel therapies.

Previous studies have shown that memory scores are associated with reduced synaptic density,(Masliah et al., 2001, 1994; Scheff et al., 2013, 2011, 2006, 1990; Scheff and Price, 1998; Sun et al., 2014) but this research was mostly conducted post-mortem in AD patients who were in late disease stages. Synaptic protein levels can also be assessed *in vivo* in cerebrospinal fluid (CSF). So far, studies have focused on few or single synaptic markers in CSF. For example, neurogranin (NRGN) is a widely studied postsynaptic protein, showing higher levels with worse memory performance in prodromal AD(Headley et al., 2018), although contradicting results have been found for memory associations in cognitively normal (CN) individuals(Dulewicz et al., 2020; Headley et al., 2018; Kvartsberg et al., 2015) and in AD dementia(Nilsson et al., 2021; Tarawneh et al., 2016). Associations of synaptosomal-associated protein 25 (SNAP25) and neuromodulin (also called growth-associated protein 43, GAP43) with memory functioning depended on disease stage, with associations in early but not in later AD.(Pereira et al., 2020) Many additional synaptic proteins exist, for which it is largely unknown if they correlate with memory functioning in the early stages of AD before dementia onset. Furthermore, individuals can have mild cognitive impairment (MCI) with *normal* amyloid (i.e., non-AD MCI), and it remains unclear what mechanisms may explain impaired memory in such individuals.

Using a proteomics approach of CSF, we aimed to analyse which synaptic proteins are related to memory scores in older non-demented individuals. We hypothesized that synaptic protein levels in CSF would be associated with worse memory scores, and that a subset of these associations would be specific for AD pathology and/or depend on cognitive stage. In two independent cohorts, we analysed cross-sectional associations of synaptic protein levels in CSF with memory scores in preclinical AD and prodromal AD, and in CN amyloid normal controls and non-AD MCI, to test if memory associations were specific for AD pathology.

## 2. Methods

### 2.1 Study participants

Two independent study cohorts, the European Medical Information Network Alzheimer’s disease multi-modal biomarker discovery study (EMIF-AD MBD)(Bos et al., 2018) and Alzheimer’s Disease Neuroimaging Initiative (ADNI) were used in these analyses. All participants provided informed consent to participate in these studies. We selected individuals who were CN and individuals with a clinical diagnosis of MCI if they had memory test scores and CSF proteomic data available. In EMIF-AD MBD, CN was defined in all centres based on neuropsychological examination scores within 1.5 standard deviation (SD) of age-, sex- and education adjusted standards, and in four centres with additional criteria (see(Bos et al., 2018) for more details). MCI was defined using cohort-specific criteria (in all cohorts Petersen criteria(Petersen, 2004) except for Lausanne, where Winblad criteria(Winblad et al., 2004) were used and Clinical Dementia Rating (CDR) of 0.5).

The ADNI (adni.loni.usc.edu) was launched in 2003 as a public-private partnership, led by Principal Investigator Michael W. Weiner, MD. The primary goal of ADNI has been to test whether serial magnetic resonance imaging (MRI), positron emission tomography (PET), other biological markers, and clinical and neuropsychological assessment can be combined to measure the progression of mild cognitive impairment (MCI) and early Alzheimer’s disease (AD). For up-to-date information, see www.adni-info.org. Detailed information about the definitions of CN and MCI in ADNI, and methodology of the memory testing can be found in the general procedures manual of ADNI(“ADNI General Procedures Manual,” 2006). In short, CN was defined as absence of memory complaints, normal scores on the Logical Memory II subscale (delayed Paragraph Recall) from the Wechsler Memory Scaled – Revised, Mini-Mental State Examination (MMSE) score ≥24 and CDR of 0. MCI was defined by a memory complaint, abnormal scores on the Logical Memory II subscale, MMSE ≥ 24 and CDR of 0.5.

For the analyses, we characterized participants from EMIF-AD MBD and ADNI into four diagnostic groups depending on their clinical and amyloid status: CN individuals with normal amyloid (controls), CN individuals with abnormal amyloid (preclinical AD), MCI individuals with normal amyloid (non-AD MCI) and MCI individuals with abnormal amyloid (prodromal AD).

### 2.2 CSF amyloid, t-tau and p-tau biomarkers

For EMIF-AD MBD, we used cut-points for amyloid status that were previously determined with gaussian mixture modelling for each center (Tijms et al., 2018). T-tau and p-tau were measured in local laboratories using either Innotest (Fujirebio Europe, Gent, Belgium) (4 cohorts), or with INNO-BIA AlzBio3 (Fujirebio Europe), and we used local cut-offs to determine abnormality(Bos et al., 2018). In ADNI, CSF amyloid, t-tau and p-tau were measured using multiplex xMAP Luminex platform (Luminex Corp, Austin, TX) with INNO-BIA AlzBio3 (Fujirebio Europe), with abnormality defined as amyloid < 192 pg/ml, t-tau > 93 pg/ml and p-tau > 23 pg/ml.

### 2.3 Proteomics

In EMIF-AD MBD, proteomics was performed with the tandem mass tag (TMT) technique using 10+1 plexing(Bos et al., 2018) as previously described(Batth et al., 2014; Magdalinou et al., 2017; Tijms, 2020). In addition, in a central laboratory (Gothenburg University, Sweden), levels of neurofilament light (NEFL) were determined with the NF-Light assay (UmanDiagnostics, Umeå, Sweden) and NRGN with an in-house immunoassay(Portelius et al., 2015). All protein levels were natural log-transformed. In ADNI, proteomic data were generated using LC/MS-MRM (panel developed by Caprion Proteome Inc.)(“MRM Assays : Caprion,” n.d.) and the Rules-Based Medicine (RBM)(“Myriad RBM Operational Procedures White Paper - Myriad RBM,” n.d.) platform. We used the pre-processed and quality checked normalized data publicly available on the ADNI website (adni.loni.usc.edu). We natural log-transformed and Z-scored individual RBM measurements and MRM peptide measurements. Next, we generated protein scores by averaging MRM peptides and correlating RBM proteins that were mapped to the same protein (r at least 0.5). Protein levels in both datasets were standardized relative to the mean and standard deviations from controls with normal amyloid, t-tau and p-tau.

The ADNI proteomic dataset consisted of 202 unique proteins and EMIF-AD MBD proteomics of 2537 proteins (missing values, ADNI: mean 0.3%, range 0 to 45%; EMIF-AD MBD: mean 6%, range 0 to 99%).

### 2.4 Synapse proteins

**Figure 1:**
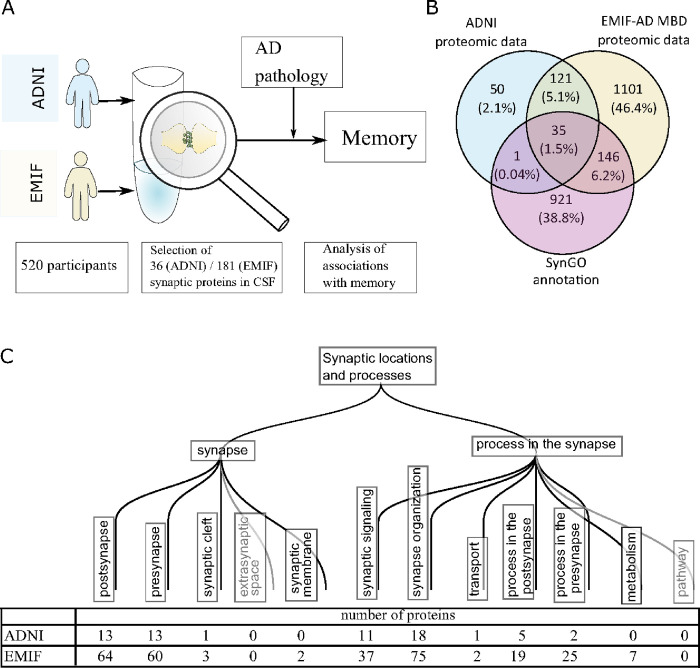
Characterization of synaptic proteomic dataset. A. Schematic overview of study design. B. Overlap of ADNI proteomics and EMIF-AD MBD proteomics with SynGO annotation used to select synaptic proteins. C. SynGO locations and processes for which synaptic proteins were included in the datasets. One protein can have multiple annotations for different locations and processes. The SynGO annotation uses a hierarchical organisation, and we show here the first and second level of subprocesses, with proteins in lower subprocesses categorized with the respective second level subprocess.

To select synaptic proteins from our proteomic datasets, we used Synaptic Gene Ontologies (SynGO) (Koopmans et al., 2019; “SynGO - Synaptic Gene Ontologies and annotations,” n.d.), a curated database with detailed annotation of known synapse-related proteins. An additional criterion for inclusion in these analyses was that proteins were measured in at least 25 individuals per diagnostic subgroup. In ADNI, 36 synaptic proteins (17% out of 202 total, missing values: mean 0.02%, range 0–0.37%) were selected, and in EMIF-AD MBD 181 proteins (13% out of 1350 total, missing values: mean 12%, range 0–66%), with an overlap of 35 proteins (missing values: mean 2.6%, range 0–31%) (Figure 1).

### 2.5 Memory tests

We measured memory functioning with both immediate and delayed recall scores of memory tests, because they may differ in their sensitivity to detect decline in different stages of AD(Bilgel et al., 2014; Jutten et al., 2021; Timmers et al., 2019). In EMIF-AD MBD, memory function was assessed with cohort specific tests (auditory verbal learning test (AVLT): 3 cohorts; Consortium to Establish a Registry for Alzheimer’s Disease wordlist (CERAD), Free and Cued Selective Reminding Test(FCSRT) and RI-48 in the remaining 3 cohorts). Test scores were Z transformed according to age, sex and education norms (as detailed in(Bos et al., 2018)), and values ranged between −5 to 5.

In ADNI, we used the Rey auditory verbal learning test (RAVLT, immediate recall: sum of correctly recalled words over 5 trials, range 0–75; delayed recall: correctly recalled words after 30-minute delay, range 0–15). We transformed the memory scores into age-, education- and sex-adjusted Z-scores, using adjustment factors for age, education and sex that were estimated from linear models for memory performance of all ADNI controls.

### 2.6 Statistical analyses

Differences in demographic variables between groups were assessed with chi-square test, Mann-Whitney U test, Student’s t-test or Kruskal-Wallis test when appropriate. Unless indicated otherwise, effects were considered significant at a p-value < 0.05. We first tested if protein levels differed depending on diagnostic group with Analysis of variance (ANOVA), and if so, performed two-sided t-tests between the diagnostic groups. To account for the multiple comparisons (in total 6 tests), we considered differences between the diagnostic groups significant at a false discovery rate (FDR)-adjusted p-value < 0.05. Next, we tested associations between memory test scores (outcome) and synaptic protein level (predictor) with linear models that included the covariates diagnostic group, age, sex and years of education (model 1) and additionally the interaction between protein level and diagnostic group (model 2). To report the effects of protein levels on memory scores across diagnostic groups, we selected proteins with a diagnostic group-interaction p-value ≥ 0.1. To report the effects of protein levels on memory scores that depended on diagnostic group, we selected proteins if they had a significant interaction with diagnostic group (interaction p-value < 0.1), and calculated associations with memory scores separately in the different diagnostic groups. For reference, we also provide statistical details of both models for all proteins (Supplementary Table 1). We then tested if the memory-associated proteins in our analyses were enriched for specific synaptic functions with the SynGO website(“SynGO - Synaptic Gene Ontologies and annotations,” n.d.), and selected biological process ontology terms for the enrichment analyses when they included at least 3 genes. For visualisation purposes, we calculated composite scores of synaptic proteins, by averaging proteins which showed an interaction (interaction p-value < 0.1) with diagnostic group on immediate or delayed recall scores. All analyses were run in R 4.1.2 “Bird Hippie”. *Emmeans* 1.4.2 was used for estimation of regression coefficients.

## 3. Results

We included 137 controls, 105 individuals with preclinical AD, 183 individuals with prodromal AD and 95 individuals with non-AD MCI (Table 1). In both studies, the preclinical and prodromal AD groups had more Apolipoprotein E (*APOE*) ε4 carriers and tended to have higher CSF t-tau and p-tau levels than controls. Compared to EMIF-AD MBD participants, ADNI participants were older (all groups), had received longer education (all groups) and had lower memory test scores (controls, preclinical and prodromal AD) (Table 1, Supplementary Figure 1).

**Figure 2:**
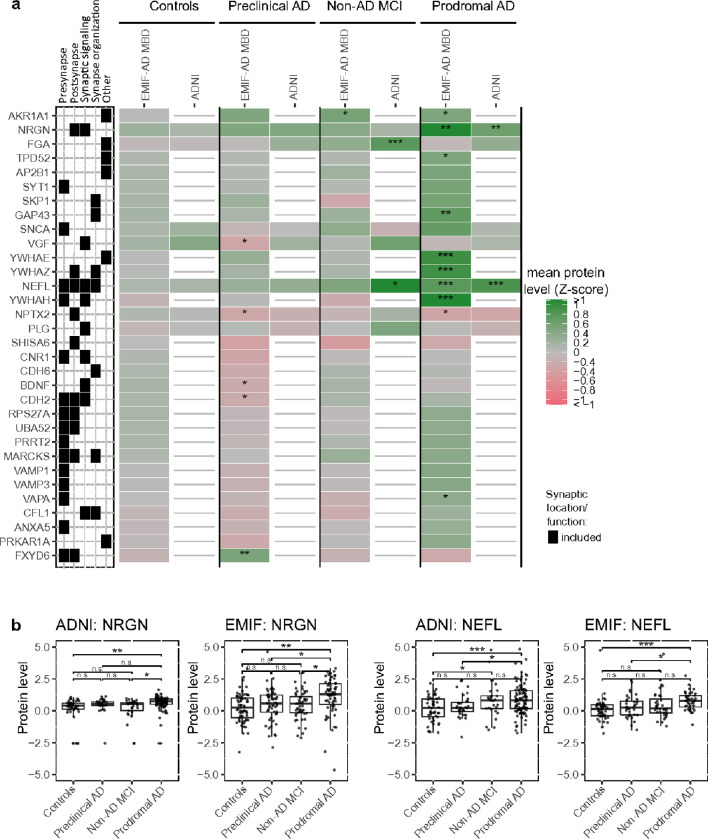
Differences in synaptic proteins depending on diagnostic group. (a) Mean protein levels are shown for top proteins depending on amyloid and cognitive subgroups, (b) boxplots of protein levels between diagnostic groups for neurogranin (NRGN) and neurofilament light (NEFL). The box of the boxplot indicates 25^th^ percentile, median and 75^th^ percentile, whiskers indicate 1.5x interquartile range. See Supplementary Table 2 for mean differences between groups and full synaptic annotation for all proteins. Synaptic category ‘Other’ refers to other synaptic locations and functions as detailed in Figure 1c. *, ** and *** indicate significant difference between diagnostic group and controls (a), or between indicated diagnostic groups (b): *, p-value < 0.05, **, p-value < 0.01; ***, p-value < 0.001, n.s.: not significant.

We selected 181 synaptic proteins in EMIF-AD MBD and 36 synaptic proteins in ADNI (Figure 1). In EMIF-AD MBD, 30 of these proteins differed between diagnostic groups (ANOVA p-value < 0.05). Relative to controls, individuals with preclinical AD showed lower levels of neurosecretory protein VGF (VGF), neuronal pentraxin-2 (NPTX2), brain-derived neurotrophic factor (BDNF) and cadherin-2 (CDH2) and higher levels of FXYD domain containing ion transport regulator 6 (FXYD6). Compared to controls, individuals with prodromal AD showed lower levels of NPTX2 and higher levels of 14-3-3 epsilon (YWHAE), 14-3-3 eta (YWHAH), 14-3-3 zeta (YWHAZ), NEFL, NRGN, GAP43, Tumor protein D52 (TPD52), VAMP-associated protein A (VAPA) and Aldo-keto reductase family 1 member A1 (AKR1A1), and the levels of these proteins were typically also higher in prodromal AD compared to preclinical AD and non-AD MCI (Figure 2A, Supplementary table 1). There was no specific association with synaptic locations (pre- and post-synapse) or synaptic functions (synaptic signalling or organization). In ADNI, only four proteins showed different levels between diagnostic subgroups (Figure 2A, Supplementary table 1). As in EMIF-AD MBD, NRGN and NEFL were increased in prodromal AD compared to controls, and compared to preclinical AD or non-AD MCI (Figure 2B). Fibrinogen alpha chain (FGA) and plasminogen (PLG) were increased in non-AD MCI relative to prodromal AD and/or controls.

### 3.1 Associations of synaptic protein levels with memory scores

**Figure 3:**
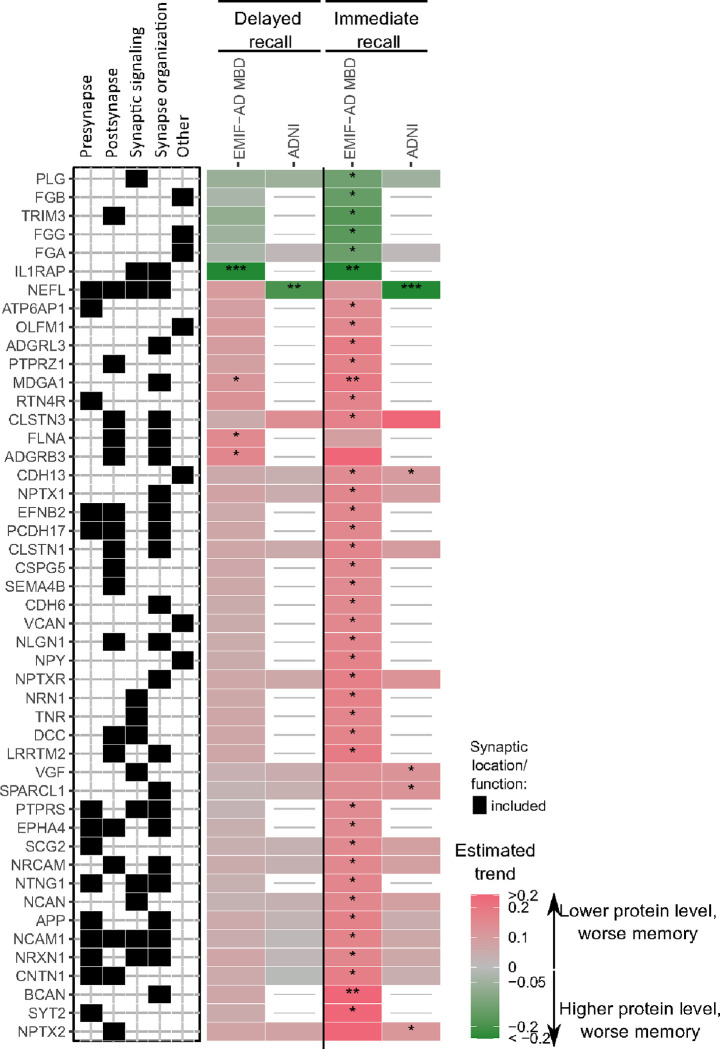
Synaptic proteins associated with memory independent of diagnostic group. Top proteins associated with memory across all diagnostic groups are shown. Associations were considered significant when proteins did not show an interaction with diagnostic group (interaction p-value ≥ 0.1) and had an association with memory function (*, p-value < 0.05; **, p-value < 0.01), ***, p-value < 0.001). Associations of all analysed synaptic proteins are provided in Supplementary table 1.

**Figure 4:**
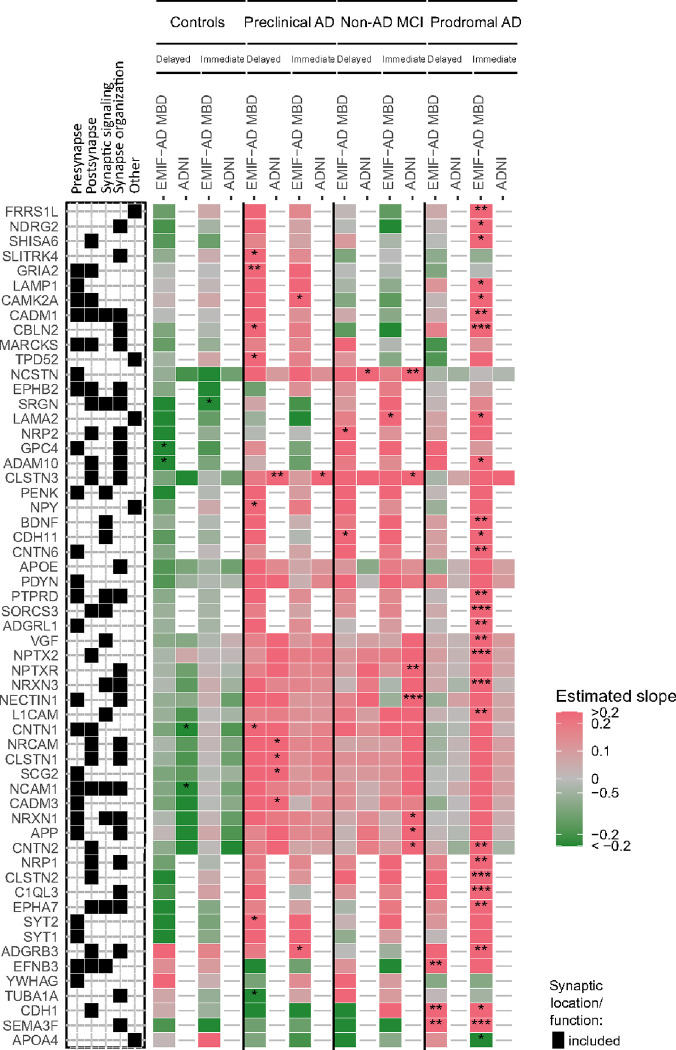
Memory-associated synaptic proteins stratified for amyloid and cognitive status. Associations between synaptic protein levels and delayed and immediate recall on word learning tests are shown stratified for diagnostic groups based on amyloid and cognitive status in EMIF-AD MBD and ADNI. Proteins which were related to memory function depending on diagnostic group in at least one cohort are shown. Associations were considered significant when proteins showed an interaction with diagnostic group on memory scores (p-value < 0.1) and showed an effect on memory function in diagnostic group-stratified analyses (p-value < 0.05). *, **, ***: significant effect of protein level on memory function in diagnostic group-stratified analyses (*, p-value < 0.05, **, p-value < 0.01; ***, p-value < 0.001). Synaptic category ‘Other’ refers to other synaptic locations and functions as detailed in Figure 1c. Associations of all analysed synaptic proteins are provided in Supplementary table 1.

Here, we will summarise the results separately for immediate and delayed recall on memory tests, and separately report associations across diagnostic groups vs. associations that depended on diagnostic group (for full results, see Supplementary table 1, and kwesenhagen.shinyapps.io/Synaptic_protein_associations_with_memory). First, for immediate recall on memory tests, we observed most associations with protein levels across all individuals (EMIF-AD MBD: 55 synaptic proteins; ADNI: 7 proteins, i.e., NEFL, SPARC-like protein 1 (SPARCL1), VGF, NPTX2, cadherin-13 (CDH13)). For most of these proteins lower levels were associated with lower immediate memory scores (EMIF-AD MBD: 49 of 55 proteins (89%); ADNI: 6 of 7 proteins, i.e., SPARCL1, VGF, NPTX2, CDH13; 86%; Figure 3). In EMIF-AD MBD, higher levels of the remaining 6 of these 55 proteins (IL-1 receptor accessory protein (IL1RAP), tripartite motif-containing protein 3 (TRIM3), FGA, fibrinogen beta and - gamma (FGB, FGG), PLG) and in ADNI, higher levels of NEFL were associated with worse immediate recall. Comparing EMIF-AD MBD and ADNI, only CDH13 was reproduced in both cohorts. We then analysed if associations between protein levels and immediate recall scores depended on diagnostic group. In EMIF-AD MBD, 30 synaptic proteins showed an interaction with diagnostic group on immediate recall scores. Of these, lower levels of 27 proteins and higher levels of one protein were associated with worse immediate recall in prodromal AD (Figure 4), while only 1 to 2 associations were observed in controls, preclinical AD and non-AD MCI (Figure 4, Supplementary table 2). In ADNI, 8 proteins showed an interaction with diagnostic group on immediate recall. For 7 of these proteins lower levels were associated with worse immediate recall in non-AD MCI (nectin-1 (NECTIN1), nicastrin (NCSTN), CLSTN3, neuronal pentraxin receptor (NPTXR), CNTN2, neurexin-1 (NRXN1), amyloid precursor protein (APP); Figure 4).

For delayed recall, 4 proteins in EMIF-AD MBD and 1 protein in ADNI were associated with worse delayed recall independent of diagnostic group (EMIF-AD MBD: IL1RAP, MAM domain-containing protein 3 (MDGA1), adhesion G protein-coupled receptor B3 (ADGRB3), filamin-A (FLNA); ADNI: NEFL; Figure 3, Supplementary table 2). For 22 proteins in EMIF-AD MBD, the association between synapse protein level and delayed recall differed between diagnostic groups. In controls, higher levels of glypican-4 (GPC4) and ADAM 10 (ADAM10) were associated with lower recall scores. In preclinical AD, lower levels of 7 proteins were associated with lower delayed recall scores, and in prodromal AD, lower levels of semaphorin-3F (SEMA3F), cadherin-1 (CDH1) and ephrin-B3 (EFNB3) were associated with lower delayed recall scores. For 11 proteins in ADNI, the association between synapse protein level and delayed recall differed between diagnostic groups. In preclinical AD, lower levels of calsyntenin-1 (CLSTN1), calsyntenin-3 (CLSTN3), secretogranin (SCG2), cell adhesion molecule 3 (CADM3) and neuronal cell adhesion molecule (NRCAM) were associated with lower delayed recall scores (Figure 4). In non-AD MCI and prodromal AD, lower levels of 2 to 3 proteins were associated with worse memory (Figure 4, Supplementary Figure 2).

**Figure 5:**
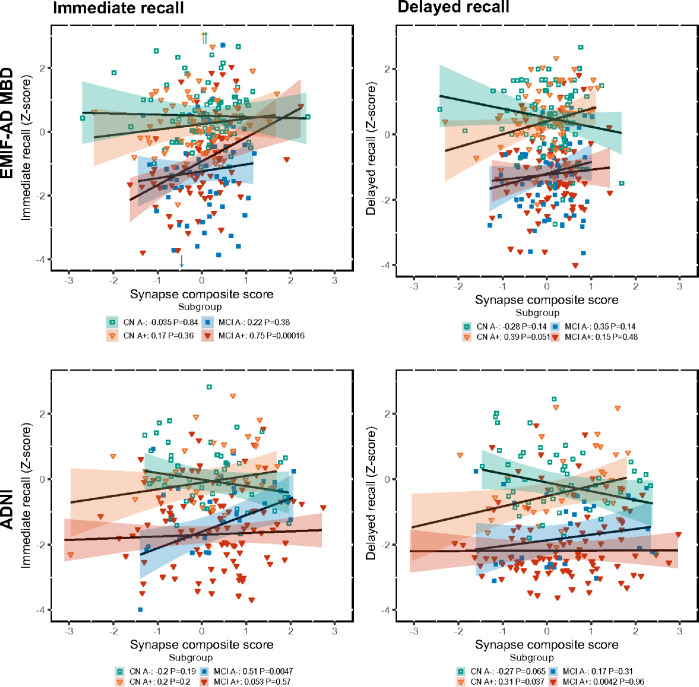
Associations of synaptic composite scores with immediate and delayed memory scores. Synaptic composites contain proteins associated with delayed recall (left) or immediate recall (right). CN A−, controls; CN A+, preclinical AD, MCI A−, non-AD MCI, MCI A+, prodromal AD.

We finally performed enrichment analyses on proteins of which the association with memory scores differed between diagnostic groups. Synaptic proteins that were associated with immediate recall in non-AD MCI showed enrichment for synapse organization, and proteins related with immediate recall in prodromal AD showed enrichment for synapse organization and trans-synaptic signalling (Supplementary Figure 2). Synaptic proteins related with delayed recall in preclinical AD showed enrichment for synapse organization and presynaptic functions (Supplementary Figure 3). Figure 5 summarizes the associations of synaptic proteins with memory scores through composite scores of synaptic proteins associated with delayed and immediate recall in EMIF-AD MBD and ADNI.

## 4. Discussion

Our main finding is that synaptic protein levels in CSF were associated with memory function in predementia AD. The synaptic proteins that related to memory function across all groups may reflect synapse loss due to other causes than AD, while proteins associated with memory in AD specifically may reflect early disease-related synapse impairment. Therefore, treatment targeting synapses may be beneficial in early phases of AD.

### 4.1 Reduced synaptic protein levels associated with worse memory functioning in preclinical AD

We found that the concentrations of five synaptic proteins (VGF, cadherin-2 (CDH2), NPTX2, BDNF and FXYD6) were reduced in preclinical AD relative to controls in EMIF-AD MBD. Reduced synaptic protein levels in CSF have been reported before in preclinical AD(Lleó et al., 2019). We extended previous studies by showing that lower memory function (i.e. delayed recall scores) associated with decreased synaptic protein levels in CSF in preclinical AD. These associations may reflect synapse loss (Masliah et al., 2001, 1994; Scheff et al., 2013, 2011, 2006, 1990; Scheff and Price, 1998; Sun et al., 2014). The deterioration of synapses could in turn result in memory impairment (Arendt, 2009; Colom-Cadena et al., 2020; Tzioras et al., 2023). Alternatively, this association can be bidirectionally interpreted, with higher levels of these proteins correlating with improved delayed recall scores. As the associated proteins did not show actual decreased levels compared to controls, it remains a possibility that higher protein levels could compensate for neuronal loss.

### 4.2 Associations of synaptic proteins with immediate and delayed recall in prodromal AD

We found higher levels of NRGN in prodromal AD relative to controls which is in line with previous studies that showed that higher NRGN levels are associated with advanced disease stage and worse cognition(Casaletto et al., 2017; Kvartsberg et al., 2015; Liu et al., 2020). In our study, however, there was only a slight trend towards a relationship between increased NRGN levels and memory function, which could imply that NRGN also relates to other aspects such as disease severity. In line with our results, decreases in NPTX2 and VGF have previously been associated with worse cognition in non-demented individuals with AD and conversion to dementia (Libiger et al., 2021; Llano et al., 2023; Llano et al., 2019). These results reinforce the prognostic value of these proteins in AD. Overall, the associations of synaptic proteins with immediate and delayed recall were similar, but substantially more associations with immediate recall than delayed recall were found in prodromal AD subjects. This may be due to floor effects in delayed recall scores.(Bilgel et al., 2014; Jutten et al., 2021) However, in our data, floor effects were not apparent in delayed recall scores (Supplementary Figure 1). A possible alternative mechanism, although only speculative, could be that these associations are specific for immediate recall through still unknown biological mechanisms, which needs further study. These proteins could be a potential target for disease modifying therapies if these proteins do reveal to be specific to immediate recall.

### 4.3 Associations of synaptic proteins with immediate and delayed recall in non-AD MCI

Of 11 memory-associated proteins in non-AD MCI, only two proteins (laminin subunit alpha-2 (LAMA2) and Calsyntenin 3 (CLSTN3)) overlapped between prodromal AD and non-AD MCI. CLSTN3 also overlapped between preclinical AD and non-AD MCI. These proteins may reflect synaptic components related to memory dysfunction that are not specific for an abnormal amyloid status. The majority of memory-associated proteins in non-AD MCI (7 proteins) were specifically related to memory in non-AD MCI. Therefore, these may reflect synaptic dysfunction and memory decline caused by different disorders. For example, for diseases such as Parkinson’s disease (PD) and dementia with Lewy bodies (DLB) that can underly non-AD MCI(Ciafone et al., 2020; Martinez-Horta and Kulisevsky, 2019), aggregated alpha synuclein could induce synaptic disturbances(Gcwensa et al., 2021; Trudler et al., 2021).

### 4.4 Associations of synaptic proteins with delayed recall in controls

Rather surprisingly, in controls a higher concentration of 5 synaptic proteins were associated with worse delayed recall (Figure 4). These proteins were not associated with memory in any of the other diagnostic groups. Some controls in EMIF-AD MBD had increased tau levels, which may have contributed to low memory scores in these individuals, as tau can impair synaptic plasticity and cause synaptic damage (Hu et al., n.d.; Kaniyappan et al., 2017; Ondrejcak et al., 2019; Piacentini et al., 2017). However, the proteins that did associate with memory in the other diagnostic groups showed associations of lower levels with lower memory scores. This suggests that the synaptic processes leading to impaired memory may differ in controls and Alzheimer’s disease.

### 4.5 Difference in associations between ADNI and EMIF-AD MBD

In general, CSF proteins showed similar associations with memory scores when comparing the EMIF-AD MBD and ADNI datasets. An exception was prodromal AD, in which individuals from the EMIF-AD MBD cohort showed many associations of lower synaptic protein levels and lower immediate recall, which were not observed in ADNI. Potentially, the lack of reproducibility between the cohorts is due to the different word learning tests used in EMIF-AD MBD and ADNI. Prodromal AD individuals in ADNI also had on average lower delayed and immediate recall scores compared to EMIF-AD MBD (Table 1), likely because in ADNI impairment on a memory test was an inclusion criterion for the prodromal AD group, which was not the case in EMIF-AD MBD. The difference in disease severity in prodromal AD and the number of proteins between both cohorts may also explain the lack of reproducibility.

### 4.6 Memory associations of synaptic subcomponents and functions

In general, we observed that synaptic components (the pre- and post-synapse) and functions (synaptic signaling) showed similar memory associations. This suggests that generalized synapse loss underlies the associations of the observed synaptic proteins with memory. However, in preclinical AD, we observed enrichment of synapse organization among proteins that were associated with delayed recall in both cohorts. This could imply synapse (re-)organization might be a process involved in memory functioning in very early AD.

### 4.7 Strengths and limitations

A limitation of this study is that cohorts used different memory tests, procedures to normalize memory scores, different criteria for the diagnostic groups, and different proteomic platforms. Nonetheless, we found similar associations of synaptic protein levels with delayed recall in both cohorts, which indicated these results are robust for cohort-dependent effects. For associations of the proteins with immediate recall in prodromal AD and non-AD MCI, we observed more heterogeneity between cohorts, perhaps reflecting that individuals with MCI form a heterogenous group. This stresses the importance of new, larger studies in individuals with prodromal AD.

Our data included only a part of the synaptic proteome (16.5%), so future studies should target a larger part of the synaptic proteome to investigate the role of synapses in memory functioning in more detail. Furthermore, while CSF proteomics allow simultaneous measurement of many proteins, it is not possible to determine whether alterations in concentrations are specific to particular anatomical brain structures. As such, future studies combining CSF proteomics and synapse PET would provide great anatomical detail of alterations in synaptic density. Lastly, the associations with memory performance we reported were not corrected for multiple testing. Instead, we tested associations with memory performance in two independent datasets which improves the robustness of the results. Strengths of our study are a relatively large sample and publication of the memory associations of the analyzed synaptic proteins in an interactive online database (available at kwesenhagen.shinyapps.io/Synaptic_protein_associations_with_memory).

### 4.8 Conclusions

Lower levels of synaptic proteins were associated with memory loss in early AD This indicates that the synapse may be an attractive target for therapeutic modulation in early AD. Further studies should therefore aim to study longitudinal relationships across different stages of AD.

## Data Availability

ADNI data can be downloaded from adni.loni.usc.edu. The raw proteomic data from EMIF-AD MBD has been submitted to the ProteomeXchange Consortium through the PRIDE partner repository, under the dataset identifier https://doi.org/10.6019/PXD019910. Requests for access to other EMIF-AD MBD data should be directed to the authors. Data sharing restrictions may apply due to consent agreements from participants in each cohort and European GDPR regulations, which limit data sharing with several non-European countries. Statistical data are provided in the supplementary information files.

## Research in Context

1. **Systematic Review:** We reviewed the literature on the associations of synaptic protein levels in cerebrospinal fluid (CSF) with memory function in Alzheimer’s disease (AD). Most studies, referenced in the text, showed memory associations for single or few synaptic markers such as neurogranin. We extend these studies by investigating memory associations of a large group of 182 synaptic proteins in CSF, and investigate if associations depend on clinical stage and amyloid status.
2. **Interpretation:** We found that reduced synaptic protein levels relate to lower memory function already in preclinical AD, suggesting synapse-targeting treatment approaches may benefit in very early phases of the disease.
3. **Future directions:** While we measured more synaptic proteins than in previous studies, this was still just a fraction of the total synaptic proteome. Future studies should perform targeted techniques to cover more of the synaptic proteome.

## Highlights

- The precise role of specific synaptic proteins in memory functioning is unclear
- We studied how 182 CSF synaptic proteins relate to memory functioning in two cohorts
- Memory function was tested in cognitively normal individuals and MCI participants
- Lower levels of 39 proteins were associated with lower memory in preclinical or prodromal AD

## Abbreviations

AD: Alzheimer’s disease
ADAM10: ADAM 10
ADGRB3: adhesion G protein-coupled receptor B3
ADNI: Alzheimer’s Disease Neuroimaging Initiative
AKR1A1: Aldo-keto reductase family 1 member A1
ANOVA: Analysis of variance
*APOE*: Apolipoprotein E
APP: amyloid precursor protein
AVLT: auditory verbal learning test
BDNF: brain-derived neurotrophic factor
CADM3: cell adhesion molecule 3
CDH1: cadherin-1
CDH2: cadherin-2
CDH11: cadherin-11
CDH13: cadherin-13
CDR: Clinical Dementia Rating
CERAD: Consortium to Establish a Registry for Alzheimer’s Disease wordlist
CLSTN1: calsyntenin-1
CLSTN3: calsyntenin-3
CN: cognitively normal
CNTN1: contactin-1
CNTN2: contactin-2
CSF: cerebrospinal fluid
DLB: dementia with Lewy bodies
EFNB3: ephrin-B3
EMIF-AD MBD: European Medical Information Network Alzheimer’s disease multi-modal biomarker discovery study
FCSRT: Free and Cued Selective Reminding Test
FDR: false discovery rate
FGA: fibrinogen alpha chain
FGB: fibrinogen beta chain
FGG: fibrinogen gamma chain
FLNA: filamin-A
FXYD6: FXYD domain containing ion transport regulator 6
GAP43: neuromodulin (also known as growth-associated protein 43)
GPC4: glypican-4
GRIA4: glutamate receptor 4
IL1RAP: IL-1 receptor accessory protein
LAMA2: laminin subunit alpha-2
LC/MS-MRM: liquid chromatography-mass spectrometry with multiple reaction monitoring
LRRTM2: leucine-rich repeat transmembrane neuronal protein 2
MCI: mild cognitive impairment
MDGA1: MAM domain-containing protein 3
MMSE: Mini-Mental State Examination
MRI: magnetic resonance imaging
NCSTN: nicastrin
NECTIN1: nectin-1
NEFL: neurofilament light
NLGN2: neuroligin-2
non-AD MCI: MCI with normal amyloid
NPTX2: neuronal pentraxin-2
NPTXR: neuronal pentraxin receptor
NRCAM: neuronal cell adhesion molecule
NRGN: neurogranin
NRXN1: neurexin-1
NRXN3: neurexin-3
PD: Parkinson’s disease
PET: positron emission tomography
PLG: plasminogen
RAVLT: Rey auditory verbal learning test
RBM: Rules-Based Medicine
SCG2: secretogranin-2
SEMA3F: Semaphorin-3F
SNAP25: synaptosomal-associated protein 25
SPARCL1: SPARC-like protein 1
SynGO: Synaptic Gene Ontologies
TMT: tandem mass tag
TPD52: tumor protein D52
TRIM3: tripartite motif-containing protein 3
VAPA: VAMP-associated protein A
VGF: neurosecretory protein VGF
YWHAE: 14-3-3 epsilon
YWHAH: 14-3-3 eta
YWHAZ: 14-3-3 zeta
